# Factors influencing prenatal and postpartum depression in Korea: a prospective cohort study

**DOI:** 10.4069/kjwhn.2021.11.17

**Published:** 2021-12-13

**Authors:** Hyeji Yoo, Sukhee Ahn, Seyeon Park, Jisoon Kim, Jiwon Oh, Minseon Koh

**Affiliations:** 1 College of Nursing, Chungnam National University, Daejeon, Korea; 2 Department of Nursing, College of Health and Welfare, Woosong University, Daejeon, Korea; 3 Department of Nursing, Yeoju Institute of Technology, Yeoju, Korea

**Keywords:** Cohort studies, Depression, Postnatal, Pregnancy, Risk factors

## Abstract

**Purpose:**

This study explored the prevalence of prenatal and postpartum depression in Korea and its influencing factors from 20 weeks of pregnancy to 12 weeks postpartum.

**Methods:**

Using a prospective cohort study design, data on women’s depression and its influencing factors were collected at 20, 28, and 36 weeks of pregnancy and at 2, 6, and 12 weeks postpartum. The participants were 219 women and 181 spouses during pregnancy; and 183 mothers and 130 spouses after childbirth. Depressive symptoms were assessed by the Edinburgh Postnatal Depression Scale and influencing factors were measured by the Postpartum Depression Predictors Inventory-Revised, parity, and spousal depression.

**Results:**

The prevalence of maternal depression was 10.5% to 21.5% before birth, and it was 22.4% to 32.8% postpartum. The prevalence slightly decreased during the prenatal period but peaked at 2 weeks postpartum. Antenatal depression was influenced by low socioeconomic status, lower self-esteem, having experienced prenatal depression, having experienced prenatal anxiety, a previous history of depression, lower social support, lower marital satisfaction, and higher life stress. The factors influencing postpartum depression were lower self-esteem, having experienced prenatal depression, having experienced prenatal anxiety, lower social support, lower marital satisfaction, and higher life stress, as well as infant temperament and maternal blues. Parity and spousal depression had no impacts.

**Conclusion:**

The prevalence and influencing factors of maternal depression changed over time. Nurses need to screen women accordingly during the perinatal period and should provide education or counseling to prevent depression and promote adjustment to parenthood.

## Introduction

Depression is a common health concern among women during pregnancy and delivery, along with fatigue and anxiety [1], and it negatively affects all family members including the spouse and children [2-4]. Thus, there is a need for a longitudinal study exploring depression and associated factors across the prenatal and postnatal periods to prevent depression, identify intervention time points, and suggest intervention content.

Beck [5], who developed the theory of postpartum depression, identified 13 predictors of postpartum depression, including 10 predictors from the prenatal period and three predictors from the postnatal period, in a study of women with postpartum depression [6]. The prenatal predictors included marital status, socioeconomic status, self-esteem, prenatal depression, prenatal anxiety, pregnancy intention, history of depression, social support, marriage satisfaction, and stress, and the postnatal predictors were child care stress, infant temperament, and maternal blues [6]. According to a study that explored the effects of predictors of postnatal depression on postnatal depression [7], an experience of depression in the prenatal period and a history of depression were relevant factors at 6 weeks postpartum, an experience of depression in the prenatal period was relevant at 4 months postpartum, and low socioeconomic status, a history of depression, and an unintended pregnancy were relevant at 6 to 9 months postpartum. In a study from South Korea (hereafter Korea) that applied the above predictors of postnatal depression [8], prenatal depression, low social support, depressed mood postpartum, and child care stress were significant factors at 2 weeks postpartum, and prenatal depression, low social support, depressed mood postpartum, and unintended pregnancy were significant factors at 6 weeks postpartum [8]. In other studies, everyday stress and child care stress [3,9-11] or stress related to interpersonal relationships or finances during pregnancy [12] influenced postpartum depression. Overall, previous research has consistently shown that pregnancy and postpartum depression are related to different types of stress.

The 10 prenatal predictors suggested by Beck et al. [6] were found to be useful indicators to identify prenatal depression. Prenatal depression has been identified as an independent predictor of postnatal depression in multiple studies [4,7-9,13-15]. Compared to healthy women, women who experienced prenatal depression had lower self-esteem, social support, and marriage satisfaction, everyday life stress, unintended pregnancy, experience of prenatal pregnancy and anxiety, history of depression, and low socioeconomic status [16]. In another study, those with prenatal depression reported lower income and insufficient social support [17]. There were more single women, women with low quality of life, subjective health status, and marriage satisfaction, and women with high stress scores in the high-risk group for prenatal depression [15,18]. Therefore, prenatal and postnatal predictors are useful indicators to identify risk of prenatal and postnatal depression and measure its impact.

Parity was also identified as a significant factor in prenatal and postnatal depression [1,5,14,16,18,19], and its effect varied by time period. During pregnancy, depression among multiparous women was higher than that among nulliparous women [1,15,16,18], but in the postpartum period, depression among nulliparous women was higher than that among multiparous women [14,19]. Spousal depression also impacted women’s prenatal and postnatal depression. Spousal depression during the prenatal period had a continuing influence on the prenatal depression of pregnant women and on their postnatal depression at 1, 3, and 6 months postpartum. Spousal depression during the postnatal period had a lagged effect on women’s depression in the following time period [4].

A limitation in the existing literature is that most studies evaluated depression at specific time points during the prenatal or postnatal periods and explored predictors of depression cross-sectionally, making it difficult to understand the onset and trajectory of depression longitudinally. However, since women’s depression fluctuates and presents in various forms as they experience the transitions from the prenatal and postnatal periods to parenthood [4,20,21], it is necessary to assess the factors that influence the onset of depression from pregnancy to postpartum and to identify the time points for evaluation and intervention. Moreover, a prospective cohort study design provides the opportunity to explore predictors at different time points, as well as predictors that remain consistent or change during the study period, which can be helpful in establishing an effective health management strategy for pregnant women’s successful transition to parenthood.

In Korea, the level of interest in prenatal depression is relatively low; instead, most studies have explored postnatal depression and its predictors among postpartum women [3,9,13,14,22]. One study investigated the prevalence of prenatal and postnatal depression and associated factors from early pregnancy to 4 weeks postpartum in a cohort of pregnant women [15]. However, prospective cohort studies analyzing prenatal and postnatal depression in women have not generally included their husbands’ depression as a factor. In a previous Korean study exploring time points in a cohort, the prevalence of depression slightly decreased from 19.3% at 12 weeks of pregnancy and 13.8% at 24 weeks of pregnancy to 14.0% at 36 weeks of pregnancy, although it then increased to 16.8% at 4 weeks postpartum [15]. In a study on postpartum depression [8], the prevalence was higher, 36.3% at 2 weeks postpartum and 36.7% at 6 weeks postpartum. International studies measured prenatal depression at 14, 24, and 34 weeks of pregnancy [1]; depression of pregnant women in the third trimester of pregnancy and at 1, 3, and 6 months postpartum [4]; and postpartum depression at 6 weeks, 4 months, and 6 to 9 months postpartum with the second trimester of pregnancy as the baseline [6]. As women adjust to pregnancy over the course of the gestational period, this study aimed to explore levels of prenatal depression at 8-week intervals beginning at 20 weeks of pregnancy (with follow-ups at 28 weeks and 36 weeks of pregnancy) and levels of postnatal depression at 2, 6, and 12 weeks postpartum.

This study aimed to identify levels of depression longitudinally at six time points from 20 weeks of pregnancy to 12 weeks postpartum among Korean women who experienced pregnancy and delivery and to identify the factors that affected depression at each time point. The specific goals were as follows.

1) To identify the prevalence of prenatal depression at 20, 28, and 36 weeks of pregnancy and the prevalence of postnatal depression at 2, 6, and 12 weeks postpartum.

2) To explore the factors that influence the prevalence of prenatal depression at 20, 28, and 36 weeks of pregnancy and the prevalence of postnatal depression at 2, 6, and 12 weeks postpartum.

## Methods

Ethics statement: This study was approved by the Institutional Review Board of Chungnam National University (No. 2-1046881-A-N-01-201704-HR-008-01-04). Informed consent was obtained from the participants.

### Study design

This study used a prospective cohort study design with pregnant women and their spouses to identify the prevalence of depression among women at six different time points during pregnancy and postpartum and explore the factors that influence depression. This study report followed the STROBE (Strengthening the Reporting of Observational Studies in Epidemiology) reporting guidelines (https://www.strobe-statement.org/).

### Setting/data collection

Participant recruitment and data collection occurred over 2 years, from 2017 to 2019. The study team cooperated with the head of the hospital and the nursing department of an obstetric hospital in a large city to conduct the study and was assigned a space in the outpatient clinic. The study team explained the aim and methods of the study using the study description form to pregnant women visiting the hospital for prenatal care and their spouses, and women and their spouses provided written consent if they wished to participate in the study. Self-report surveys were distributed to the women and their spouses when they were at 20 weeks of pregnancy to collect data. The data collection method in each time period of the cohort study has been described elsewhere in detail [23].

### Participants

The participants of this study were pregnant women receiving prenatal care at two women’s hospitals in Daejeon Korea, and their spouses. The inclusion criteria were women with a singleton pregnancy of 20 to 24 weeks and their spouses. The inclusion and exclusion criteria have been described in a previous publication in detail [23].

### Measurement tools

#### Depression

The Korean Edinburgh Postnatal Depression Scale (EPDS) [24], which is based on the EPDS [25] and validated among Korean pregnant women was used, to measure depression in pregnant women and their spouses at each time point. This tool includes 10 items measured on a 4-point scale and was validated in both prenatal and postnatal women and their spouses [26,27]. Cox et al. suggested a cutoff of 12/13 or 9/10 in the postnatal period depending on the use [25]. The validation study of the Korean EPDS reported a cutoff of 9/10 [20], and the same cutoff was most appropriate for men [27]. Therefore, 9/10 was set as the cutoff. The reliability of this scale was reported in a previous study [23].

#### Factors associated with depression

This study used the Postpartum Depression Predictors Inventory (PDPI) [28], translated into Korean and validated in Korea [29], to measure the factors associated with depression at each time point. To measure 10 prenatal factors, this instrument contained one item on marital status, one item on socioeconomic status, three items on self-esteem, one item on experience of prenatal depression, one item on experience of prenatal anxiety, four items on pregnancy intention, one item on history of depression, 16 items on social support, three items on marriage satisfaction, and seven items on everyday life stress. To measure three postnatal factors, there were three items on child care stress, three items on infant temperament, and one item on maternal blues. In the validation study for the Korean PDPI [29], support from women’s side of the family and men’s side of the family was measured separately, according to Korean culture, which resulted in added items to the family support category. Therefore, the number of items in the PDPI was 39. Marital status (married vs. other), socioeconomic status (high/middle vs. low), experience of prenatal depression (yes/no), experience of prenatal anxiety (yes/no), and maternal blues (yes/no) were measured as nominal variables. The sums of each item score (yes=1, no=0) for the remaining variables (self-esteem, pregnancy intention, social support, marriage satisfaction, everyday life stress, child care stress, and infant temperament) were measured as continuous variables. The risk associated with the postpartum depression predictors was considered to be high when the score was high for the continuous variables.

#### General characteristics and characteristics associated with delivery

The general characteristics of participants in this study included women’s age, education level, occupation, marital status, and household income as prenatal factors and parity and delivery type as postnatal factors. Additional information about the women, their spouses, and the newborns has been reported in a previous study [23].

### Variables

Questionnaire items in the measurement tools were variables including the participants’ characteristics.

### Bias

There was no potential source of bias in selecting participants.

### Study size

The sample size was calculated using the odds ratio (OR) of postpartum depression for predictors of postpartum depression [29]. Among the 13 predictors of postpartum depression, the OR of pregnancy intention for postpartum depression was 1.57 [29]. When the sample size was calculated using the two-sided test, an OR of 1.57, an effect size of .14, a significance level of .05, and a power of .80 in the G*Power 3.1.9 program, the minimum sample size was 180. Considering the dropout rate from a previous prospective cohort study with pregnant women [23], this study recruited 225 pairs at baseline with the aim to recruit 140% of the minimum sample size and secure 180 pairs of pregnant women and their spouses at 12 weeks postpartum. Participants who responded at two or more prenatal data collection points and two or more postnatal data collection points were included in the final sample. The final analytic sample included 219 pregnant women and 181 spouses and 183 postpartum women and 130 spouses.

### Data analysis

The collected data were analyzed using IBM SPSS ver. 24.0 (IBM Corp., Armonk, NY, USA). Women’s general and pregnancy-related characteristics were explored through frequency and descriptive analyses. The levels of prenatal depression, postnatal depression, and related factors among women at each time period were explored through frequency and descriptive analyses. The factors that impacted prenatal depression and postnatal depression in women were analyzed using multiple logistic regression at each time period. As the pattern of participants’ dropout was not consistent, it was difficult to consider the samples at each time point as a single sample. Therefore, a longitudinal analysis was not conducted.

## Results

### General and delivery-related characteristics of women

The average age of the pregnant women who participated in the prenatal survey was 32.9 (±3.41). Most women (91.3%) had graduated college or higher, and majority (55.3%) were employed. All participants were married (100%), and a majority (53.0%) reported a monthly household income of greater than 4,000,000 Korean won. Among the pregnant women who participated in the postnatal survey, 68.3% were nulliparous, and the majority (60.1%) had a vaginal delivery (Table 1).

### Prevalence of depression and distribution of factors associated with depression among women

The prevalence of depression among women was 21.5% at 20 weeks, 20.5% at 28 weeks, and 10.5% at 36 weeks of pregnancy, and it was 32.8% at 2 weeks, 24.6% at 6 weeks, and 22.4% at 12 weeks postpartum. Nine prenatal factors, three postnatal factors, parity, and spousal depression were identified as factors associated with depression.

Among the nine prenatal factors, 6.4% to 8.7% of women in the prenatal period and 8.2% to 10.4% of women in the postnatal period were classified as low socioeconomic status. The score for a lack of self-esteem was 0.37 to 0.44 points in the prenatal period and 0.38 to 0.48 in the postnatal period. Experiences of prenatal depression were reported by 66.2% to 68.9%, experiences of postnatal depression by 52.5% to 53.6%, experiences of prenatal anxiety by 66.2% to 68.9%, and experiences of postnatal anxiety by 44.8% to 48.1%. The score for unintended pregnancy was 0.75 to 0.80 points in the prenatal period and 0.73 to 0.77 points in the postnatal period, while a history of depression was present in 12.3% to 14.2% of participants in the prenatal period and 12.6% to 15.3% in the postnatal period. The scores for a lack of social support were 2.05 to 2.24 and 2.33 to 2.96 points, those for marriage dissatisfaction were 0.17 to 0.24 and 0.25 to 0.37 points, and those for everyday life stress were 0.51 to 0.82 and 0.37 to 0.42 points in the prenatal period and in the postnatal period, respectively. Among the three postnatal factors, the scores for child care stress ranged from 0.46 to 0.94 points, those for infant temperament ranged from 0.62 to 0.95 points, and 41.5% to 48.1% of participants reported maternal blues. Among the pregnant participants, 68.0% were nulliparous, while 69.3% of the postpartum participants were nulliparous. The prevalence of depression among spouses was 10.5% at 20 weeks, 10.5% at 28 weeks, and 12.7% at 36 weeks of pregnancy and 6.2% at 2 weeks, 10.0% at 6 weeks, and 6.9% at 12 weeks postpartum (Table 2).

### Factors that influenced prenatal and postpartum depression among women

The impact of nine prenatal factors, parity, and spousal depression on prenatal depression was evaluated at 20 weeks, 28 weeks, and 36 weeks of pregnancy. Eight factors consistently had a significant effect on prenatal depression at the three time points. These were low socioeconomic status, lack of self-esteem, experience of prenatal depression, experience of prenatal anxiety, history of depression, lack of social support, marriage dissatisfaction, and everyday life stress. Unintended pregnancy was significantly associated with prenatal depression at 28 weeks and 36 weeks of pregnancy. However, at every time point, parity and prenatal depression among spouses were not associated with prenatal depression among

At 20 weeks of pregnancy, a history of depression (OR, 5.40), low socioeconomic status (OR, 4.89), low marriage satisfaction (OR, 2.90), experience of prenatal anxiety (OR, 2.60), experience of prenatal depression (OR, 2.48), lower self-esteem (OR, 2.48), higher everyday life stress (OR, 1.60), and lower social support (OR, 1.30) were associated with prenatal depression. At 28 weeks of pregnancy, low socioeconomic status (OR, 3.38), experience of prenatal anxiety (OR, 3.02), history of depression (OR, 2.99), lower self-esteem (OR, 2.69), lower marriage satisfaction (OR, 2.54), experience of prenatal depression (OR, 2.12), higher everyday life stress (OR, 1.88), lower social support (OR, 1.39), and lower intention for pregnancy (OR, 1.34) were associated with prenatal depression. At 36 weeks of pregnancy, low socioeconomic status (OR, 11.81), experience of prenatal anxiety (OR, 9.88), experience of prenatal depression (OR, 4.58), history of depression (OR, 4.97), lower self-esteem (OR, 3.12), lower marriage satisfaction (OR, 2.51), lower intention for pregnancy (OR, 1.73), higher everyday life stress (OR, 1.69), and lower social support (OR, 1.32) were associated with prenatal depression (Table 3).

As for the factors that influenced postnatal depression, the impact of nine prenatal factors, three postnatal factors, parity, and spousal depression on women’s depression was evaluated at 2 weeks, 6 weeks, and 12 weeks postpartum. Among the prenatal factors, experience of prenatal depression, experience of prenatal anxiety, low self-esteem, social support, marriage satisfaction, and high everyday life stress were associated with postnatal depression at the three time points. Among the postnatal factors, infant’s temperament and maternal blues were continuously significant. At 2 weeks postpartum, unintended pregnancy and, at 12 weeks postpartum, low socioeconomic status were additional factors that were associated with depression among women at each time point. At all time points, history of depression, parity, and spousal depression were not associated with postpartum depression among women.

At 2 weeks postpartum, maternal blues (OR, 7.82), higher child care stress (OR, 2.89), and more difficult infant temperament (OR, 1.95) were associated with postnatal depression among postnatal factors, and experience of prenatal anxiety (OR, 3.13), lower marriage satisfaction (OR, 2.51), experience of prenatal depression (OR, 2.39), higher everyday life stress (OR, 2.30), lower self-esteem (OR, 1.53), lower intention for pregnancy (OR, 1.40), and lower social support (OR, 1.29) were associated with postpartum depression among prenatal factors. At 6 weeks postpartum, higher child care stress (OR, 2.66), maternal blues (OR, 2.43), and more difficult infant temperament (OR, 1.83) were associated with postpartum depression among postnatal factors. Among prenatal factors, lower marriage satisfaction (OR, 3.14), higher everyday life stress (OR, 2.34), experience of prenatal depression (OR, 2.06), experience of prenatal anxiety (OR, 2.00), lower self-esteem (OR, 1.86), and lower social support (OR, 1.22) were associated with postpartum depression. At 12 weeks postpartum, maternal blues (OR, 3.21), higher child care stress (OR, 1.73), and more difficult infant temperament (OR, 1.58) were associated with postpartum depression among postnatal factors, and among prenatal factors, low socioeconomic status (OR, 6.14), experience of prenatal depression (OR, 4.21), lower self-esteem and marriage satisfaction (OR, 3.34), experience of prenatal anxiety (OR, 2.71), higher everyday life stress (OR, 2.34), and lower social support (OR, 1.23) were associated with postpartum depression (Table 4).

## Discussion

Depression among women constantly fluctuated in different patterns during the prenatal and postnatal time periods. The prevalence of depression among women decreased to 10.5% at 36 weeks of pregnancy from 20.5% to 21.5% at 20 weeks and 28 weeks of pregnancy. This result is similar to a previous report that prevalence of prenatal depression decreased slightly from 16 weeks of pregnancy to 25 weeks and 36 weeks using the same instrument, but with a cutoff of 12 [26]. The reason could be that toward the end of pregnancy, women adapt to the physical and psychological changes from pregnancy and have increased anticipation of meeting the baby.

During the postpartum period, the prevalence of depression was highest at 32.8% at 2 weeks postpartum, which decreased to 22.4%–24.6% with time. The reasons for high prevalence in the early postpartum period at 2 weeks seem to be that as women’s main task becomes performing the role of a mother to care for the infant, they experience child care stress, lack of confidence in their role [22], or maternal blues. Compared to previous longitudinal studies that followed depression prevalence from pregnancy to postpartum [1,4-6,15,30], this study showed a significant difference in depression prevalence. In a previous cohort with pregnant women [15] that evaluated depression using the same tool and cutoff [15], prenatal depression was reported at a similar level as in the current study at 13.9% to 19.4%, but the prevalence at 4 weeks postpartum was 16.7%, which is lower than the prevalence of depression at 2 weeks and 6 weeks postpartum in this study. Among pregnant women in Finland [30], the prevalence of depression measured using Center for Epidemiologic Studies Depression Scale (C-ESD) was 11.0% at the third trimester of pregnancy and 10.5% at 3 months postpartum, showing a similar trend across prenatal and postnatal periods. A study that evaluated depression using the EPDS (cutoff score of 12) or C-ESD among pregnant women in the United States [4] reported a slightly higher prevalence of 38.5% in the third trimester of pregnancy, but a consistently lower prevalence of 23.1% at 1 month postpartum and 19.2% at 3 months postpartum. These differences could be due to the sociocultural characteristics related to pregnancy, delivery, and postpartum in the countries where participants live or the use of varying measurement time points and tool cutoffs.

Low socioeconomic status, low self-esteem, experience of prenatal depression, experience of prenatal anxiety, history of depression, low social support, low marriage satisfaction, and high everyday life stress among pregnant women were factors associated with prenatal depression at all three time points. Pregnancy intention was an additional factor at 28 weeks and 36 weeks of pregnancy. This result is similar to previous studies that found prenatal depression to be associated with income level [15,17,18, 21], history of depression, plans to become pregnant, stress levels [15,18], marriage satisfaction [18], social support [17], prenatal anxiety, self-esteem, and unemployment [16]. Various psychosocial stresses in the lives of women and their families accumulate during pregnancy and constantly influence prenatal depression along with the stress from pregnancy [12]. Therefore, it is necessary to inform women about effective coping strategies for stress during pregnancy. A previous study reported that depression was decreased among pregnant women by providing prenatal education intervention about physical and psychological adaptation, delivery preparation, infant care, and spousal relationships [31], which suggests directions for interventions for perinatal women and their families. Nurses can evaluate the factors associated with prenatal depression before and after pregnant women’s prenatal health checks and satisfy women’s and family members’ psychosocial needs through education and counseling. Moreover, in order to reduce the stress about delivery and burden related to child care, it would be possible to provide education on delivery preparation including understanding the delivery process, practicing breathing methods, and visiting the delivery room, along with education and role exercise opportunities about breastfeeding and infant care.

Maternal blues, difficult infant temperament, and child care stress were identified as factors associated with postnatal depression, consistent with previous studies [8]. It seems that the risk of postpartum depression is increased difficulties in adjusting during the transition to parenthood including mood changes in the early postpartum period, lack of understanding of infant temperaments, and decreased maternal confidence due to difficulties in child care. Therefore, in order to reduce the rate of postpartum depression, practical education and information resources related to infant care should be provided during mothers’ stay in the postnatal ward or recovery centers. Most prenatal factors that influenced prenatal depression were significantly associated with postnatal depression. This result is similar to factors identified in previous studies, such as prenatal depression [7,8,13], prenatal anxiety [7], everyday life stress [7,28], socioeconomic status [7], lack of social support [7,8,28], and financial stress [12]. When stressors during pregnancy are not resolved and continue postpartum, these factors become long-term and consistent stressors [7,30]. Unlike a study that found pregnancy intention to be a significant factor associated with depression among pregnant women measured 6 weeks and 6 to 9 months postpartum [7], intention for pregnancy influenced depression in women at 28 weeks and 36 weeks of pregnancy and at 2 weeks postpartum in this study. Intention for pregnancy is related to the acceptance of pregnancy; therefore, women who do not accept their pregnancy may negatively receive the physical and psychological discomfort of pregnancy [32]. Moreover, the compounded effect of unintended pregnancy and socioeconomic burden could have resulted in the significant relationships between unintended pregnancy and prenatal depression and between low socioeconomic status and postpartum depression. However, the finding that everyday life stress, social support, marriage satisfaction, and child care stress were common factors associated with prenatal and postpartum depression could suggest the possibility of family-centered prevention interventions for postpartum depression that aid the adjustment of women and their family members, including their spouses. Education about the recovery of mothers and the parental role for mothers who visit for their regular postnatal checkups, online or phone education and counseling for mothers and family members who cannot travel, and service linkage to local mental health welfare centers can provide opportunities to participate in postpartum depression prevention programs.

Parity was not significantly associated with depression among women. This finding is consistent with studies that found the number of children not to be associated with prenatal depression [15,18], suggesting the importance of nursing practices that assess and intervene for women’s depression regardless of their parity. However, this result diverges from studies that reported that prenatal depression was higher among multiparous mothers [1,15,16,18], postpartum depression was higher among nulliparous mothers [14,19], and the severity of postpartum depression was twice as high when mothers with consistently high levels of depression had children [30]. Further exploration of the association between prenatal and postpartum depression and parity is necessary. Depression among spouses also was not associated with prenatal or postpartum depression. This result is not consistent with a previous study that found early postpartum depression among spouses impacted women’s depression up until 6 months postpartum [4]. A reason for this could be the gap between expectations about gender roles and parenting roles as husband and wife and the actual execution of those roles, as well as differences in the characteristics of spousal relationships by country.

This study has a few limitations. Caution is required in generalizing the results of this study since the sample was constructed using convenience sampling of pregnant women who visited a women’s hospital in one location. Moreover, during the 10-month data collection from 20 weeks of pregnancy to 12 weeks postpartum, the dropout rates were 20% at 36 weeks of pregnancy and 27% at 2 weeks postpartum due to various reasons such as change in participants’ residence, health issues of the child, and loss to follow-up. Effective strategies to minimize dropout, such as frequent contact during the transition from the prenatal to postnatal period and postcards to congratulate women on their new baby, are thus necessary.

In summary, the prevalence of depression among women tracked from 20 weeks of pregnancy to 12 weeks postpartum was higher during postpartum (22.4%–32.8%) than during pregnancy (10.5%–21.5%). Common factors associated with prenatal depression were low socioeconomic status, experience of prenatal depression, experience of prenatal anxiety, history of depression, low self-esteem, low social support, low marriage satisfaction, and high everyday life stress. Common factors associated with postpartum depression were high child care stress, difficult temperament of infant, and maternal blues, in addition to the prenatal factors. Therefore, nurses should regularly assess the level of depression and factors associated with depression from the prenatal period to postpartum, provide interventions to address depression that are in line with pregnant women’s and their families’ needs, and support healthy pregnancy, delivery, and postpartum adaptation.

## Figures and Tables

**Table 1.** Demographic and obstetric characteristics of the female participants

Variable	Categories	n (%) or mean±SD
Prenatal period (N=219)		
Age (year)		32.9±3.41
Education	High school or less	19 (8.7)
	University or above	200 (91.3)
Job	Yes	121 (55.3)
	No	98 (44.7)
Marital status	Married	219 (100)
Monthly family income (KRW)	<2 million	13 (5.9)
	2–3.99 million	90 (41.1)
	≥4 million	116 (53.0)
Postpartum period (N=183)		
First-time mother	Yes	125 (68.3)
	No	58 (31.7)
Type of birth	Vaginal birth	110 (60.1)
	Cesarean birth	73 (39.9)

KRW: Korean won (1 million KRW is approximately 900 US dollars).

**Table 2.** Changes in factors influencing women’s depression from the prenatal to postpartum period

Variable	Categories	Possible range	n (%) or mean±SD					
			Prenatal period (N=219)			Postpartum period (N=183)		
			20 weeks	28 weeks	36 weeks	2 weeks	6 weeks	12 weeks
Prenatal factor								
Socioeconomic status	Low vs. middle/high		19(8.7)	16(7.3)	14(6.4)	17(9.3)	15(8.2)	19(10.4)
Self-esteem		0–3	0.44±0.70	0.42±0.71	0.37±0.65	0.46±0.84	0.38±0.76	0.48±0.87
Prenatal depression experience	Yes		150(68.5)	151(68.9)	145(66.2)	96(52.5)	98(53.6)	97(53.0)
	No							
Prenatal anxiety experience	Yes		138(63.0)	122(55.7)	137(62.6)	88(48.1)	82(44.8)	86(47.0)
	No							
Pregnancy intention		0–4	0.79±1.12	0.80±1.10	0.75±1.08	0.76±1.07	0.73±1.05	0.77±1.07
History of previous depression	Yes		31(14.2)	28(12.8)	27(12.3)	28(15.3)	27(14.8)	23(12.6)
	No							
Social support		0–16	2.24±3.15	2.05±3.05	2.17±3.11	2.47±3.40	2.33±3.41	2.96±3.91
Marital satisfaction		0–3	0.24±0.55	0.18±0.51	0.17±0.52	0.27±0.59	0.25±0.58	0.37±0.73
Life stress		0–7	0.82±0.94	0.58±0.97	0.51±0.98	0.42±0.70	0.37±0.64	0.42±0.79
Postpartum factor								
Parenting stress		0–3				0.94±0.82	0.90±0.81	0.46±0.69
Infant temperament		0–3				0.62±0.87	0.95±1.07	0.64±1.02
Maternal blues	Yes					76(41.5)	88(48.1)	85(46.4)
	No							
								

**Table 3.** Factors influencing women’s prenatal depression during pregnancy (N=219)

Variable	Prenatal period								
	20 weeks			28 weeks			36 weeks		
	OR	*p*	95% CI	OR	*p*	95% CI	OR	*p*	95% CI
Prenatal factor									
Socioeconomic status^†^	4.89	.001	1.84–12.81	3.38	.023	1.18–9.64	11.81	<.001	3.68–37.88
Self-esteem	2.48	<.001	1.59–3.87	2.69	<.001	1.72–4.21	3.12	<.001	1.90–5.12
Prenatal depression experience^†^	2.48	.007	1.29–4.79	2.12	.042	1.03–4.38	4.58	<.001	1.96–10.70
Prenatal anxiety experience^†^	2.60	.014	1.21–5.57	3.02	.003	1.44–6.34	9.88	.002	2.26–43.27
Pregnancy intention	1.21	.164	0.92–1.60	1.34	.040	1.01–1.78	1.73	.003	1.20–2.48
Previous history of depression^†^	5.40	<.001	2.42–12.06	2.99	.011	1.29–6.95	4.97	.001	1.87–13.24
Social support	1.30	<.001	1.17–1.44	1.39	<.001	1.24–1.56	1.32	<.001	1.17–1.49
Marital satisfaction	2.90	<.001	1.68–5.02	2.54	.001	1.44–4.46	2.51	.002	1.41–4.46
Life stress	1.60	.005	1.15–2.22	1.88	<.001	1.34–2.66	1.69	.003	1.20–2.39
Parity^†^	0.70	.295	0.36-1.37	0.64	.197	0.32–1.26	0.47	.090	0.20–1.13
Paternal depression^†^ (n=181)^‡^	1.22	.737	0.38-3.96	0.86	.820	0.24–3.15	1.67	.451	0.44–6.39

OR: Odds ratio; CI: confidence interval.

† The reference groups were as follows: socioeconomic status (low), prenatal depression (yes), prenatal anxiety (yes), previous history of depression (yes), parity (first-time mother), and paternal depression (depressive).

‡ In this analysis, the maternal sample size was matched with the paternal sample size.

**Table 4.** Factors influencing women’s postpartum depression during the postpartum period (N=183)

Variable	Postpartum period								
	2 weeks			6 weeks			12 weeks		
	OR	*p*	95% CI	OR	*p*	95% CI	OR	*p*	95% CI
Prenatal factor									
Socioeconomic status^†^	2.54	.070	0.93–6.95	1.13	.846	0.34–3.73	6.14	<.001	2.28–16.58
Self-esteem	1.53	.020	1.07–2.19	1.86	.003	1.23–2.80	3.34	<.001	2.15–5.18
Prenatal depression experience^†^	2.39	.008	1.26–4.55	2.06	.044	1.02–4.16	4.21	.001	1.87–9.47
Prenatal anxiety experience^†^	3.13	.001	1.64–5.97	2.00	.046	1.01–3.96	2.71	.007	1.31–5.61
Pregnancy intention	1.40	.019	1.06–1.86	1.14	.429	0.83–1.55	1.31	.089	0.96–1.78
Previous history of depression^†^	1.67	.221	0.74–3.81	1.67	.257	0.69–4.03	2.05	.134	0.80–5.25
Social support	1.29	<.001	1.16–1.44	1.22	<.001	1.10–1.34	1.23	<.001	1.13–1.35
Marital satisfaction	2.51	.002	1.42–4.47	3.14	<.001	1.74–5.66	3.34	<.001	2.02–5.54
Life stress	2.30	.001	1.43–3.70	2.34	.001	1.43–3.84	2.34	<.001	1.48–3.72
Postpartum factor									
Child care stress	2.89	<.001	1.87–4.46	2.66	<.001	1.68–4.22	1.73	.022	1.09–2.76
Infant temperament	1.95	<.001	1.35–2.81	1.83	<.001	1.34–2.51	1.58	.005	1.15–2.16
Maternity blues^†^	7.82	<.001	3.88–15.74	2.43	.013	1.21–4.87	3.21	.002	1.54–6.72
Parity^†^	1.27	.495	0.64–2.48	1.60	.231	0.74–3.43	1.35	.448	0.62–2.93
Paternal depression^†^ (n=130)^‡^	1.62	.526	0.37–7.15	1.36	.663	0.34–5.36	1.29	.764	0.25–6.62

OR: Odds ratio; CI: confidence interval.

† The reference groups were as follows: socioeconomic status (low), prenatal depression (yes), prenatal anxiety (yes), previous history of depression (yes), parity (first-time mother), and paternal depression (depressive).

‡ In this analysis, the maternal sample size was matched with the paternal sample size.

## References

[1] Korja R, Nolvi S, Kataja EL, Scheinin N, Junttila N, Lahtinen H (2018). The courses of maternal and paternal depressive and anxiety symptoms during the prenatal period in the FinnBrain Birth Cohort study. PLoS One.

[2] Brandão T, Brites R, Pires M, Hipólito J, Nunes O (2019). Anxiety, depression, dyadic adjustment, and attachment to the fetus in pregnancy: actor-partner interdependence mediation analysis. J Fam Psychol.

[3] Han J, Lee JS (2016). The effects of preschooler temperament and maternal postnatal depression, depression, and parenting stress on preschooler externalizing problem behavior. Korean J Child Stud.

[4] Paulson JF, Bazemore SD, Goodman JH, Leiferman JA (2016). The course and interrelationship of maternal and paternal perinatal depression. Arch Womens Ment Health.

[5] Beck CT (1993). Teetering on the edge: a substantive theory of postpartum depression. Nurs Res.

[6] Beck CT, Records K, Rice M (2006). Further development of the Postpartum Depression Predictors Inventory-Revised. J Obstet Gynecol Neonatal Nurs.

[7] Alves S, Fonseca A, Canavarro MC, Pereira M (2019). Predictive validity of the Postpartum Depression Predictors Inventory-Revised (PDPI-R): a longitudinal study with Portuguese women. Midwifery.

[8] Youn JH, Jeong IS (2013). Predictors of postpartum depression: prospective cohort study. J Korean Acad Nurs.

[9] Lee EJ, Park JS (2015). Development of a prediction model for postpartum depression: Based on the mediation effect of antepartum depression. J Korean Acad Nurs.

[10] Lee Y, Hyun MH (2019). The effects of neuroticism on postpartum depression: A dual mediating effect of gratitude and parenting stress. Stress.

[11] Reid KM, Taylor MG (2015). Social support, stress, and maternal postpartum depression: a comparison of supportive relationships. Soc Sci Res.

[12] Liu CH, Giallo R, Doan SN, Seidman LJ, Tronick E (2016). Racial and ethnic differences in prenatal life stress and postpartum depression symptoms. Arch Psychiatr Nurs.

[13] Lee CY, Cho HH (2019). Effects of breast-feeding adaptation and quality of sleep on postpartum depression in puerperal women. J Korean Soc Matern Child Health.

[14] Lee DJ, Park JS (2018). The effects of fatigue, postpartum family support on postpartum depression in postpartum women. Korean Parent Child Health J.

[15] Chung JH (2017). Prevalence and risk factor of pregnancy complications. Report No. 2016-ER-6306-01.

[16] Koh M, Ahn S, Kim J, Park S, Oh J (2019). Pregnant women’s antenatal depression and influencing factors. Korean J Women Health Nurs.

[17] Lee SH, Youk GY (2018). Factors affecting antenatal depression of pregnant women. AJMAHS.

[18] Ryu HM (2014). Prevalence and risk factors of pregnancy complications. Report No. 11-1352173-000082-01.

[19] Ministry of Health and Welfare, Ministry of Health and Welfare (2018). Report on the results of the postpartum care survey.

[20] Kim YK, Hur JW, Kim KH, Oh KS, Shin YC (2008). Clinical application of Korean version of Edinburgh Postnatal Depression Scale. J Korean Neuropsychiatr Assoc.

[21] Park JH, Karmaus W, Zhang H (2015). Prevalence of and risk factors for depressive symptoms in Korean women throughout pregnancy and in postpartum period. Asian Nurs Res (Korean Soc Nurs Sci).

[22] Kim JS (2018). The effect of parenting stress and depression and fatigue on quality of life in early postpartum mothers. J Converg Inf Technol.

[23] Yoo H, Ahn S, Oh J, Park S, Kim J, Koh M (2021). Depression and stress in Korean parents: a cohort study. Appl Nurs Res.

[24] Kim YK, Won SD, Lim HJ, Choi SH, Lee SM, Shin YC (2005). Validation study of the Korean version of Edinburgh Postnatal Depression Scale (K-EPDS). J Korea Soc Depression Bipolar Disorders.

[25] Cox JL, Holden JM, Sagovsky R (1987). Detection of postnatal depression. Development of the 10-item Edinburgh Postnatal Depression Scale. Br J Psychiatry.

[26] Lydsdottir LB, Howard LM, Olafsdottir H, Thome M, Tyrfingsson P, Sigurdsson JF (2019). The psychometric properties of the Icelandic version of the Edinburgh Postnatal Depression Scale (EPDS) when used prenatal. Midwifery.

[27] Matthey S, Barnett B, Kavanagh DJ, Howie P (2001). Validation of the Edinburgh Postnatal Depression Scale for men, and comparison of item endorsement with their partners. J Affect Disord.

[28] Records K, Rice M, Beck CT (2007). Psychometric assessment of the Postpartum Depression Predictors Inventory-Revised. J Nurs Meas.

[29] Youn JH, Jeong IS (2011). Predictive validity of the postpartum depression predictors inventory-revised. Asian Nurs Res (Korean Soc Nurs Sci).

[30] Kiviruusu O, Pietikäinen JT, Kylliäinen A (2020). Trajectories of mothers’ and fathers’ depressive symptoms from pregnancy to 24 months postpartum. J Affect Disord.

[31] Koh M, Kim J, Yoo H, Kim SA, Ahn S (2021). Development and application of a couple-centered antenatal education program in Korea. Korean J Women Health Nurs.

[32] Brunton R, Gosper K, Dryer R (2021). Psychometric evaluation of the pregnancy-related anxiety scale: Acceptance of pregnancy, avoidance, and worry about self subscales. J Affect Disord.

