# Corrigendum: Factors influencing prenatal and postpartum depression in Korea: a prospective cohort study

**DOI:** 10.4069/kjwhn.2021.11.17.e1

**Published:** 2022-02-08

**Authors:** Hyeji Yoo, Sukhee Ahn, Seyeon Park, Jisoon Kim, Jiwon Oh, Minseon Koh

**Affiliations:** 1 College of Nursing, Chungnam National University, Daejeon, Korea; 2 Department of Nursing, College of Health and Welfare, Woosong University, Daejeon, Korea; 3 Department of Nursing, Yeoju Institute of Technology, Yeoju, Korea

Korean J Women Health Nurs 2021;27(4):326-336. https://doi.org/10.4069/kjwhn.2021.11.17

This corrigendum is for correcting some errors in Tables 3 and 4. Number of subjects for variables and indicator variables was not correctly reported. A corrected Tables 3 and 4 are attached below. The authors apologize for any inconvenience that this may have caused.

**Table 3.** Factors influencing women’s prenatal depression during pregnancy (N=219)

Variable	Prenatal period								
	20 weeks			28 weeks			36 weeks		
	OR	*p*	95% CI	OR	*p*	95% CI	OR	*p*	95% CI
Prenatal factor									
Socioeconomic status^†^	4.89	.001	1.84–12.81	3.38	.023	1.18–9.64	11.81	<.001	3.68–37.88
Self-esteem	2.48	<.001	1.59–3.87	2.69	<.001	1.72–4.21	3.12	<.001	1.90–5.12
Prenatal depression experience^†^	2.48	.007	1.29–4.79	2.12	.042	1.03–4.38	4.58	<.001	1.96–10.70
Prenatal anxiety experience^†^	2.60	.014	1.21–5.57	3.02	.003	1.44–6.34	9.88	.002	2.26–43.27
Pregnancy intention	1.21	.164	0.92–1.60	1.34	.040	1.01–1.78	1.73	.003	1.20–2.48
Previous history of depression^†^	5.40	<.001	2.42–12.06	2.99	.011	1.29–6.95	4.97	.001	1.87–13.24
Social support	1.30	<.001	1.17–1.44	1.39	<.001	1.24–1.56	1.32	<.001	1.17–1.49
Marital satisfaction	2.90	<.001	1.68–5.02	2.54	.001	1.44–4.46	2.51	.002	1.41–4.46
Life stress	1.60	.005	1.15–2.22	1.88	<.001	1.34–2.66	1.69	.003	1.20–2.39
Parity^†^	0.70	.295	0.36-1.37	0.64	.197	0.32–1.26	0.47	.090	0.20–1.13
Paternal depression^†^ (n=181)^‡^	1.22	.737	0.38-3.96	0.86	.820	0.24–3.15	1.67	.451	0.44–6.39

OR: Odds ratio; CI: confidence interval.

† The indicator groups were as follows: socioeconomic status (low), prenatal depression (yes), prenatal anxiety (yes), previous history of depression (yes), parity (first-time mother), and paternal depression (depressive).

‡ In this analysis, the maternal sample size was matched with the paternal sample size.

**Table 4.** Factors influencing women’s postpartum depression during the postpartum period (N=183)

Variable	Postpartum period								
	2 weeks			6 weeks			12 weeks		
	OR	*p*	95% CI	OR	*p*	95% CI	OR	*p*	95% CI
Prenatal factor									
Socioeconomic status^†^	2.54	.070	0.93–6.95	1.13	.846	0.34–3.73	6.14	<.001	2.28–16.58
Self-esteem	1.53	.020	1.07–2.19	1.86	.003	1.23–2.80	3.34	<.001	2.15–5.18
Prenatal depression experience^†^	2.39	.008	1.26–4.55	2.06	.044	1.02–4.16	4.21	.001	1.87–9.47
Prenatal anxiety experience^†^	3.13	.001	1.64–5.97	2.00	.046	1.01–3.96	2.71	.007	1.31–5.61
Pregnancy intention	1.40	.019	1.06–1.86	1.14	.429	0.83–1.55	1.31	.089	0.96–1.78
Previous history of depression^†^	1.67	.221	0.74–3.81	1.67	.257	0.69–4.03	2.05	.134	0.80–5.25
Social support	1.29	<.001	1.16–1.44	1.22	<.001	1.10–1.34	1.23	<.001	1.13–1.35
Marital satisfaction	2.51	.002	1.42–4.47	3.14	<.001	1.74–5.66	3.34	<.001	2.02–5.54
Life stress	2.30	.001	1.43–3.70	2.34	.001	1.43–3.84	2.34	<.001	1.48–3.72
Postpartum factor									
Child care stress	2.89	<.001	1.87–4.46	2.66	<.001	1.68–4.22	1.73	.022	1.09–2.76
Infant temperament	1.95	<.001	1.35–2.81	1.83	<.001	1.34–2.51	1.58	.005	1.15–2.16
Maternity blues^†^	7.82	<.001	3.88–15.74	2.43	.013	1.21–4.87	3.21	.002	1.54–6.72
Parity^†^	1.27	.495	0.64–2.48	1.60	.231	0.74–3.43	1.35	.448	0.62–2.93
Paternal depression^†^ (n=130)^‡^	1.62	.526	0.37–7.15	1.36	.663	0.34–5.36	1.29	.764	0.25–6.62

OR: Odds ratio; CI: confidence interval.

† The indicator groups were as follows: socioeconomic status (low), prenatal depression (yes), prenatal anxiety (yes), previous history of depression (yes), maternity blues (yes), parity (first-time mother), and paternal depression (depressive).

‡ In this analysis, the maternal sample size was matched with the paternal sample size.

